# Public knowledge of cardiovascular disease and its risk factors in Kuwait: a cross-sectional survey

**DOI:** 10.1186/1471-2458-14-1131

**Published:** 2014-11-04

**Authors:** Abdelmoneim Awad, Hala Al-Nafisi

**Affiliations:** Department of Pharmacy Practice, Faculty of Pharmacy, Kuwait University, P.O. Box 24923, Safat, 13110 Kuwait

**Keywords:** Cardiovascular disease, Knowledge, Symptoms, Risk factors, Heart attack, Stroke, Kuwait

## Abstract

**Background:**

Cardiovascular disease (CVD) is estimated to cause 46% of all mortalities in Kuwait. To design effective primary and secondary prevention programs, an assessment of a population’s prior CVD knowledge is of paramount importance. There is scarcity of data on the existing CVD knowledge among the general Kuwaiti population. Hence, this study was performed to assess the level of knowledge towards CVD types, warning symptoms of heart attack or stroke, and CVD risk factors. It also explored public views on the community pharmacists’ role in CVD prevention and management.

**Methods:**

A descriptive cross-sectional survey was performed using a pretested self-administered questionnaire on a sample of 900 randomly selected Kuwaiti individuals. Descriptive and multivariate logistic regression analysis were used in data analysis.

**Results:**

The response rate was 90.7%. Respondents’ knowledge about types of CVD, heart attack or stroke symptoms was low. Almost 60% of respondents did not know any type of CVD, and coronary heart disease was the commonest identified type (29.0%). Two-fifths of participants were not aware of any heart attack symptoms, and the most commonly known were chest pain (50.4%) and shortness of breath (48.0%). Approximately half of respondents did not recognize any stroke symptoms, and the most commonly recognized were ‘confusion or trouble speaking’ (36.4%) and ‘numbness or weakness’ (34.7%). Respondents’ knowledge regarding CVD risk factors was moderate. The commonest factors identified by over four-fifths of participants were smoking, obesity, unhealthy diet and physical inactivity. In the multivariate logistic regression analysis, independent predictors of better level of CVD knowledge were females, age 50–59 years, high level of education, regular eating of healthy diet, and had a family history of CVD. Most of respondents only identified the role that pharmacists had to play is to help patients manage their medications, with a minimal role in other aspects of CVD prevention and management.

**Conclusions:**

There are deficiencies in CVD knowledge among Kuwaiti population, which could turn into insufficient preventative behaviours and suboptimal patient outcomes. There is an apparent need to establish more wide-spread and effective educational interventions, which should be sensitive to the perceptions, attitudes, and abilities of targeted individuals.

**Electronic supplementary material:**

The online version of this article (doi:10.1186/1471-2458-14-1131) contains supplementary material, which is available to authorized users.

## Background

Cardiovascular diseases (CVDs) are group of disorders that involve the heart or blood vessels or both. They include coronary heart disease (CHD), cerebrovascular disease, peripheral arterial disease, rheumatic heart disease, congenital heart disease, and deep vein thrombosis and pulmonary embolism [1]. CVDs are the leading cause of deaths in both developed and developing countries. In 2008, 30% (17.5 million people) of global all-cause mortalities were from CVDs. Of these, 6.2 and 7.3 million were due to stroke and CHD, respectively. It is expected to increase to 23.3 million by 2030 [2]. In Kuwait, CHD is the major cause of morbidity and mortality and CVDs are estimated to cause 46.0% of all mortalities [3, 4]. Kuwait is an oil producing country in the Middle East region, it has witnessed rapid economic and sociodemographic changes during the last four decades. The major transitions in the developing societies have had apparent influences on behavioural, social, and lifestyle patterns. As a consequence of these dramatic changes in Kuwait, the prevalence of hypertension is 25.3%, diabetes 23.3%, dyslipdaemia 70.3%, obesity 48.2% and smoking 17.8% [5, 6].

Cardiovascular disease (CVD) is one of the most preventable causes of death in the world, due to the fact that the majority of its risk factors are preventable or controllable, such as hypertension, dyslipidaemia, diabetes, obesity, smoking, lack of physical activity, stress, unhealthy dietary practices and diabetes. The social and environmental causes of CHD and stroke are well recognized, and enhanced population-based prevention programs could result in a significant decrease in CVD morbidity and mortality [7]. Knowledge about CVD and its modifiable risk factors is a vital pre-requisite to change the individuals’ health attitudes, behaviours and lifestyle practices [8, 9]. Knowledge improvement to the recognition of heart attack and stroke symptoms will lead to earlier presentation to medical care that may result in better patients outcomes [10–12]. Good knowledge about CVD risk factors among individuals will aid them to be proactive in decreasing their risk since the majority of the risk factors are modifiable [12–14].

The estimation of the baseline knowledge about CVD among the population has significant public health application as it helps in developing targeted educational programs [12]. Knowledge of CVD, its symptoms and risk factors have been studied worldwide in various populations. Some of these studies have focused on patients [12, 15–18], while others have studied general populations [11, 14, 19–25]. Yet, little is known about the CVD knowledge in the Eastern Mediterranean region, where only two studies were published. The first was conducted in the Gulf Cooperation Council (GCC) countries to assess the level of awareness of stroke risk factors and symptoms among the general population [19]. The second was conducted in Jordan to examine the public knowledge and awareness of CVD and its risk factors [20]. However, there are no published studies to date that comprehensively assess the existing knowledge of CVD among the public in Kuwait, where CVDs are estimated to cause 46.0% of all mortalities. Hence, this study was designed to determine the level of current CVD knowledge among Kuwaiti individuals, and to identify factors that are associated with knowledge levels. The CVD knowledge assessed included level of awareness of types of CVD, warning symptoms of heart attack or stroke, and CVD risk factors. This study also explored the public views on the role of pharmacists in prevention and management of CVD.

## Methods

A descriptive, cross-sectional survey was conducted in Kuwait, a Middle-Eastern country with an area of 17,820 km^2^ and an estimated population of 3,065,850 people; 35.6% of whom are Kuwaitis (2011 estimate) [26]. The survey was conducted during the period from January to June 2014. The study population consisted of Kuwaiti individuals from six governorates of Kuwait: Al-Ahmadi, Al-Farwaniyah, Al-Jahra, Capital, Hawalli and Mubarak Al-Kabeer. Ethical approval for this study was obtained from the “Ministry of Health Ethical Committee, Kuwait”.

The sample size was determined using PS power and sample size calculator V.3.05 [27]. A sample of 808 individuals would be necessary to determine a 10% difference in proportion between two groups; for example, male versus female with 80% power and at 5% significance level. Assuming a response rate of 90%, a sample size of 900 was selected randomly from government and private enterprises, houses and diwaniyas (local congressional meetings) of the pharmacy students families, relatives, friends and neighbours within each governorate and Kuwait University colleges. From these, individuals were contacted and given an explanation with regard to the purpose of the study. They were free to refuse participation in the study. Data were collected anonymously via self-administered questionnaire. Those who agreed to take part in the study were given the questionnaires, which were completed anonymously and collected after completion. They were assured for confidentiality and gave written consent to participate in the study. Incentives were not offered for completion of the questionnaire. Exclusion criteria were expatriates, age ≤20 years and ≥80 years, and health care professionals and students.

A literature review of previous studies regarding CVD knowledge was conducted to identify potential items for the study instrument. Based on the literature search, the study questionnaire was adapted from validated surveys that were previously used in Australia, Pakistan, North Ireland, Canada, Jordan and South Africa [11, 12, 14, 15, 20, 21]. The content validity of the adapted questionnaire were established by a research group at Kuwait University. The questionnaire was translated into Arabic and subjected to a process of forward and backward translation. The accuracy and meaning of the translated versions both forward and backward were checked, and recommended amendments where necessary were discussed before being finalized. It was pretested for content, design, readability, and comprehension on 16 Kuwaiti subjects, and modifications were made as necessary so that the questionnaire was simple to understand and answer, yet gave accurate data.

The pre-tested questionnaire consisted of four sections, and it contains both open-ended and close-ended questions (Additional file 1). The first section included seventeen items to provide information about the demographic and clinical characteristics of the respondents (age, gender, marital status, educational level, employment, residence, monthly income, personal health, height, weight, smoking status, exercise, healthy food, lifestyle, and family history of CVD). Section two consisted of four questions to provide information about the participants’ medical status including presence of chronic diseases, chronic use of medications, recent measures of blood pressure, blood cholesterol and glucose levels, and last time for checking blood pressure, body weight, blood cholesterol and blood glucose. The third section included three questions to determine the knowledge with regard to six types of CVD, five heart attack warning symptoms, five stroke warning symptoms, and nine CVD risk factors. Section four included two questions to explore the public views on the role of pharmacists in prevention and management of CVD, and their intention to use CVD prevention services if being offered in the community pharmacy.

Data were entered into the Statistical Package for Social Sciences (SPSS, version 21, SPSS, Chicago, IL, U.S.A.) and descriptive analysis was conducted. The results were reported as percentage (95% confidence interval) and median (Interquartile range). A scoring system was applied to measure the respondents’ knowledge of each of the following: six types of CVD, five heart attack warning symptoms, five stroke warning symptoms, and nine CVD risk factors. The internal consistency for each of the four sections to determine knowledge was assessed using Cronbach’s α test. The test results were as follows: respondents’ knowledge regarding types of CVD, 0.91; warning symptoms of heart attack, 0.86; warning symptoms of stroke, 0.88; and CVD risk factors, 0.88. Knowledge scores were categorized for each section as follows: knowledge regarding types of CVD: low knowledge [≤2], moderate knowledge [3, 4] and high knowledge [≥5]; knowledge in relation to warning symptoms of heart attack or stroke: low knowledge [≤2], moderate knowledge [3] and high knowledge [≥4]; and knowledge about CVD risk factors: low knowledge [≤4], moderate knowledge [6–8] and high knowledge [9]. Finally the overall CVD knowledge score was calculated as a continuous variable by summing the respondent’s scores of CVD types, heart attack symptoms, stroke symptoms and CVD risk factors; the internal consistency using Cronbach’s α was 0.92. The maximum overall knowledge score was 25: low knowledge [≤12], moderate knowledge [13–19] and high knowledge [≥20].

Univariate logistic regression was performed to determine the relationship of each independent variable with the overall knowledge of CVD. All variables with p ≤0.25 in the univariate analysis were included in the multiple logistic regression analysis to determine the factors that are independently associated with overall CVD knowledge. Only the results of multivariate logistic analysis are reported showing odds ratio (OR) and 95% confidence interval. Statistical significance was accepted at p <0.05. For each model, response options for the dependent variable were categorized as either “low knowledge” or “moderate/high knowledge”. The predictor variables were categorized as follows: (1) gender: males and females; (2) age**:** [20–29 years], [30–39 years], [40–49 years], [50–59 years], ≥60 years; (3) level of education: low-intermediate [0–12 years] for those who completed secondary school or less, and high [*>*12 years] for those who had diploma, or bachelor degree or postgraduate degree; (4) monthly income**:** low [<500 Kuwaiti Dinars (KD)], middle [500–1000 KD] and high [>1000 KD]; (5) personal health: excellent, very good, good; (6) smoking status [yes, no]; (7) self-reported weight description [underweight, normal, overweight and obese]; (8) BMI [underweight, normal, overweight and obese]; (9) 30 minutes exercise/week [0–2 times, 3–4 times and ≥5 times/week]; (10) eating healthy food: everyday and not everyday; (11) lifestyle: very stressful/stressful, relatively stressful, free from stress; (12) positive family history of CVD: yes had a family history of CVD and no family history of CVD; (12) chronic disease: yes had a chronic disease and no chronic disease.

## Results

### Study population

Table 1 shows the demographic and clinical characteristics of the study participants. A total of 900 Kuwaiti nationals were approached to be included in the study, 816 agreed to participate, giving a response rate of 90.7%. Their median (IQR) age was 33 (14) years. Of the respondents, 59.6% were females, 92.8% had high education, and 52.4% with a high monthly income of greater than 1000 KD. The median (IQR) BMI of the study population was 26.3 (6.3) kg/m^2^. Three hundred and forty nine participants (42.8%) reported that they had normal body weight; however, 32.7% of them were found to be either overweight or obese according to their calculated BMI. Approximately one-fifth (17.9%) of respondents indicated that they smoked, 13.0% indicated that they used to exercise for 30 minutes, 5 or more times/week, and 27.2% reported eating healthy food everyday. One-third (33.3%) of participants reported to have very stressful or stressful lifestyle. About one-quarter of participants indicated to have chronic diseases including hypertension, diabetes, dyslipidaemia and CHD.

**Table 1:** **Demographic and clinical characteristics of study participants (n = 816)**

Characteristics	Frequency (%)
**Gender**	
Male	330 (40.4)
Female	486 (59.6)
**Marital status**	
Single^⊥^	331 (40.6)
Married	485 (59.4)
**Age (years)**	
20-29	330 (40.4)
30-39	270 (33.1)
40-49	142 (17.4)
50-59	59 (7.2)
≥ 60	15 (1.8)
**Employment**	
Unemployed^∋^	92 (11.3)
Employed	724 (88.7)
**Educational level**	
Low-intermediate education	59 (7.2)
High education	757 (92.8)
**Residence (Governorates)**	
Capital	266 (32.6)
Hawalli	146 (17.9)
Al-Ahmadi	104 (12.7)
Mubarak Al-Kabeer	102 (12.5)
Al-Farwaniyah	101 (12.4)
Al-Jahra	97 (11.9)
**Monthly income**	
< 500 Kuwaiti Dinars (KD)	92 (11.3)
500-1000 Kuwaiti Dinars (KD)	296 (36.3)
> 1000 Kuwaiti Dinars (KD)	428 (52.4)
**Personal health**	
Excellent	376 (46.1)
Very good	343 (42.0)
Good	97 (11.9)
**Self-reported weight description**	
Under weight	30 (3.7)
Normal	349 (42.8)
Overweight	408 (50.0)
Obese	29 (3.6)
**BMI (kg/m** ^**2**^ )*^	
< 18.5	19 (2.3)
18.5-24.9	287 (35.2)
25-30	311 (38.1)
>30	198 (24.3)
**Smokers**	146 (17.9)
**30 Minutes exercise/week**	
0-2 times	598 (73.3)
3-4 times	112 (13.7)
≥ 5 times/week	106 (13.0)
**Eating Healthy food Everyday**	222 (27.2)
**Lifestyle**	
Very stressful or Stressful	272 (33.3)
Relatively stressful	443 (54.3)
Free from stress	101 (12.4)
**Family history of CVD**	269 (33.0)
**Chronic disease** ^**Φ**^	193 (23.7)
Prior Hypertension	73 (8.9)
Prior Diabetes	54 (6.6)
Prior Dyslipidaemia	111 (13.6)
Prior CHD	33 (4.0)

^⊥^ *Includes divorced and widowed.*

^∋^ *Includes retired, housewives and students.*

* *Percentages may not total 100 because of missing response.*

*^Calculated from self-reported weight and height.*

^Φ^ *Total number exceeds 193 due to multiple responses.*

All respondents who had diabetes and CHD reported taking medications for these diseases. Seventeen (23.3%) of the hypertensive patients, and 57 (51.4%) of those with dyslipidaemia admitted that they did not take medications for both diseases. Of the study population, 275 (33.7%), 367 (45%) and 343 (42%) reported that they did not know their recent measures for blood pressure, blood cholesterol and blood glucose, respectively. About one-fifth (19.7%) of respondents had never checked their blood pressure, and approximately one-quarter had never checked their blood cholesterol (27.2%) and glucose (24.4%) levels.

### CVD knowledge

Table 2 shows respondents’ knowledge about CVD types, heart attack or stroke warning symptoms, and CVD risk factors. The median (IQR) score for knowledge about the six types of CVD was 1.0 (2.0) [low knowledge]: 59.4% did not know any type of CVD, and 15.6% identified four or more types. The commonest type of CVD identified was CHD (29.0%), followed by congenital heart disease (27.8%), and deep vein thrombosis and pulmonary embolism (24.5%). The median (IQR) score for knowledge about the five heart attack symptoms was 2.0 (3.0) [low knowledge]: 40.7% did not recognize any heart attack symptom, whereas 7.2% knew one, 11.0% two, 20.0% three, 12.6% four, and 8.5% five. The commonest heart attack symptom reported was ‘chest pain or discomfort’ (50.4%) followed by ‘difficulty in breathing or shortness of breath’ (48.0%), and ‘pain or discomfort in arms or shoulder’ (41.2%). The median (IQR) score for knowledge about the five stroke symptoms was 1.0 (3.0) [low knowledge]: 47.8% did not know any stroke symptom, whereas 9.4% identified one, 15.0% two, 9.3% three, 8.1% four, and 10.4% five. The most common stroke symptoms indicated by participants were ‘sudden confusion or trouble speaking or understanding others’ (36.4%), followed by ‘sudden numbness or weakness of the face, arm, or leg’ (34.7%), and ‘sudden dizziness, trouble walking, or loss of balance or coordination’ (32.2%). Respondents’ knowledge regarding the CVD risk factors was better than that for types of CVD and symptoms of heart attack or stroke. The median (IQR) score for knowledge about the nine CVD risk factors was 7.0 (4.0) [moderate knowledge]: 6.6% did not know any CVD risk factor, whereas 13.2% indicated one to four, 32.0% five to seven, and 48.2% identified eight or nine risk factors. The commonest risk factors identified by over four-fifths of respondents were smoking, obesity, unhealthy diet and physical inactivity.

**Table 2:** **Respondents’ knowledge of CVD types, heart attack and stroke symptoms and CVD risk factors of (n = 816)**

Category	Frequency	Percentage (95% CI)
**CVD Types**		
Coronary heart disease	237	29.0 (26.0- 32.3)
Congenital heart disease	227	27.8 (24.8-31.1)
Deep vein thrombosis and Pulmonary embolism	200	24.5 (21.6-27.6)
Rheumatic heart disease	124	15.2 (12.8-17.9)
Peripheral arterial disease	110	13.5 (11.3-16.1)
Cerebrovascular disease	108	13.2 (11.0-15.8)
**Heart attack symptoms**		
Chest pain or discomfort	411	50.4 (46.9-53.9)
Difficulty in breathing or shortness of breath	392	48.0 (44.6-51.5)
Pain or discomfort in arms or shoulder	336	41.2 (37.8-44.7)
Feeling weak, light-headed, or faint	207	25.4 (22.4-28.5)
Pain or discomfort in the jaw, neck ,or back	140	17.2 (14.7-20.0)
**Stroke symptoms**		
Sudden confusion or trouble speaking or understanding others	297	36.4 (33.1-39.8)
Sudden numbness or weakness of the face, arm, or leg	283	34.7 (31.4-38.1)
Sudden dizziness, trouble walking, or loss of balance or coordination	263	32.2 (29.1-35.6)
Severe headache with no known cause	214	26.2 (23.3-29.4)
Sudden trouble seeing in one or both eyes	183	22.4 (19.6-25.5)
**CVD Risk factors**		
Smoking	724	88.7 (86.3-90.8)
Obesity	705	86.4 (83.8-88.6)
Unhealthy diet	700	85.8 (83.2- 88.1)
Physical inactivity	683	83.7 (81.0-86.1)
High LDL Cholesterol levels	569	69.7 (66.4-72.8)
Hypertension	525	64.3 (60.9-67.6)
Positive family history	511	62.6 (59.2-65.9)
Stress	506	62.0 (58.6-65.3)
Diabetes mellitus	433	53.1 (49.6-56.5)

### Factors independently associated with CVD knowledge

Overall CVD knowledge was evaluated in this study as a continuous variable. Responses to 25 questions regarding six types of CVD, five heart attack symptoms, five stroke symptoms and nine CVD risk factors were scored as correct or incorrect and each participant received one point for each correct response. The median (IQR) knowledge score of respondents was 10.0 (8.0) out of a maximum score of 25: 5.3% (n = 43) did not indicate any correct response, whereas 15.4% (n =126) listed one to six correct responses, 40.1.% (n = 327) seven to twelve, 28.8% (n =235) thirteen to nineteen, and 10.4% (n = 85) twenty to twenty five correct responses.

Table 3 shows the results of the multivariate analysis for factors associated with overall knowledge of CVD. In the univariate analysis, factors significantly associated with CVD knowledge included gender, age, education, smoking status, monthly income, eating healthy diet, self-reported weight, and family history of CVD (p <0.05). These statistically significant factors plus variables with a p-value ≤0.25 in the univariate analysis were included in the multivariate logistic regression model. As shown in Table 3, factors independently associated with knowledge were gender, age, education, eating healthy diet, and family history of CVD (p <0.05). The knowledge about CVD was significantly greater among females compared to males (p = 0.022), and those aged 50–59 years compared to other age groups (p = 0.007). The study participants were found to be more knowledgeable about CVD if they reported attending high education (p = 0.036), eating healthy diet everyday (p <0.001 ), and having a family history of CVD (p <0.001 ).

**Table 3:** **Association between overall CVD knowledge and respondents’ characteristics (n = 816)**

Characteristics	Moderate/h\igh knowledge frequency (%)	OR (95% CI)	P-value
**Gender**			0.022
Male	112 (33.9)	Reference	
Female	208 (42.8)	1.58 (1.07-2.33)	
**Marital state**			0.757
Single	120 (36.3)	0.95 (0.678-1.33)	
Married	200 (41.2)	Reference	
**Age (years)**			0.007
20-29	112 (33.9)	1.26 (0.36-4.42)	
30-39	118 (43.5)	1.72 (0.49-6.05)	
40-49	50 (35.2)	1.01 (0.29-3.53)	
50-59	35 (59.3)	3.05 (1.85-9.9)	
≥ 60	5 (33.3)	Reference	
**Employment**			0.468
Unemployed	30 (32.6)	0.80 (0.44-1.47)	
Employed	290 (40.1)	Reference	
**Educational level**			0.036
Low-intermediate education	15 (25.4)	Reference	
High education	305 (45.3)	2.01 (1.05-3.85)	
**Monthly income**			0.089
< 500 Kuwaiti Dinars (KD)	33 (35.9)	Reference	
500-1000 Kuwaiti Dinars (KD)	107 (36.1)	0.78 (0.44-1.38)	
> 1000 Kuwaiti Dinars (KD)	180 (42.1)	1.15 (0.65-2.05)	
**Self-reported weight description**			0.150
Under weight	6 (20.0)	0.69 (0.20-2.33)	
Normal	132 (37.8)	1.37 (0.59-3.15)	
Overweight	172 (42.2)	1.67 (0.74-3.80)	
Obese	10 (34.5)	Reference	
**Smoking status**			0.189
Smoker	43 (29.5)	0.73 (0.46-1.17)	
Non-smoker	277 (41.3)	Reference	
**30 Minutes exercise/week**			0.675
0-2 times	241 (40.3)	1.02 (0.62-1.67)	
3-4 times	34 (30.4)	0.82 (0.44-1.55)	
≥ 5 times/week	45 (42.5)	Reference	
**Eating Healthy food**			<0.001
Everyday	115 (51.8)	1.99 (1.38-2.88)	
Not everyday	205 (34.5)	Reference	
**Family history of CVD**			<0.001
Yes	127 (47.2)	1.54 (1.11-2.12)	
No	193 (35.3)	Reference	
**Chronic disease**			0.822
Yes	85 (44.0)	0.96 (0.65-1.40)	
No	235 (37.7)	Reference	

### Role of pharmacist in CVD prevention and management

Table 4 shows the respondents’ views on the role of community pharmacists in CVD prevention and management. Seven-in-ten responders (n = 583; 71.4%) indicated that the pharmacist, rather than the nurse, should help patients manage their medications. Almost six-in-ten responders perceived that it was the role of the nurse rather than the pharmacist to measure blood glucose (59.6%), blood cholesterol (59.3%), and blood pressure (58.7%). Over two-fifth of participants reported that it was the role of a pharmacist rather than a nurse to offer advice on smoking cessation (46.9%) and healthy diet (41.9%).

**Table 4:** **Respondents’ views on the role of community pharmacists in CVD prevention and management**

Role	Frequency	Percentage (95% CI)
**Helping the patients in managing their prescribed medicine**		
Pharmacist	583	71.4 (68.2-74.5)
Nurse	128	15.7 (13.3-18.4)
Not sure	105	12.9 (10.7-15.4)
**Offer advice on smoking cessation**		
Pharmacist	383	46.9 (43.5-50.4)
Nurse	222	27.2 (24.2-30.4)
Not sure	211	25.9 (22.9-29.0)
**Offer healthy diet advice**		
Pharmacist	342	41.9 (38.5-45.4)
Nurse	194	23.8 (20.9-26.9)
Not sure	280	34.3 (31.1-37.7)
**Offer advice on physical activity**		
Pharmacist	287	35.2 (31.9-38.6)
Nurse	276	33.8 (30.6-37.2)
Not sure	253	31.0 (27.9-34.3)
**Measure blood pressure**		
Pharmacist	210	25.7 (22.8-28.9)
Nurse	479	58.7 (55.2-62.1)
Not sure	127	15.6 (13.2-18.3)
**Measure blood glucose**		
Pharmacist	200	24.5 (21.6-27.6)
Nurse	486	59.6 (56.1-62.9)
Not sure	130	15.9 (13.5-18.7)
**Measure blood cholesterol**		
Pharmacist	182	22.3 (19.5-25.4)
Nurse	484	59.3 (55.9-62.7)
Not sure	150	18.4 (15.8-21.3)

Respondents were asked about their intention to use CVD prevention services if become available in the community pharmacy. More than three-quarters of participants reported their intention to use weight measurement (n = 705; 86.4%), blood glucose measurement (n = 688; 84.3%), blood pressure measurement (n = 678; 83.1%), blood cholesterol measurement (n = 680; 83.3%), advice on smoking cessation (n = 639; 78.3%), advice on healthy diet (n = 635; 77.8%), and advice on physical activity (n = 615; 75.4%).

## Discussion

### Key findings

This is the first known study to be conducted in Kuwait, and probably in the GCC countries to comprehensively demonstrate the current level of public knowledge about types of CVD, warning symptoms of heart attack or stroke and CVD risk factors. Over half of the study population were not aware of any type of CVD, two-fifths were not aware of any heart attack symptom, and approximately half were not aware of any stroke symptom. Despite this low knowledge about types of CVD and its symptoms, respondents were much better knowledgeable of CVD risk factors, nearly half of them were aware of eight or nine factors. The present findings would be the first step in providing a quantitative measurement of CVD knowledge and identifying specific knowledge gaps. This will aid in the assessment of the adequacy of the present community educational programs, and could be utilized in designing future targeted public health promotion campaigns to enhance CVD knowledge in the hope of improving early recognition, reducing time to treatment, and reducing the risk of CVD. Moreover, this study showed that the respondents did not recognize the roles of community pharmacists in CVD prevention and management.

### CVD knowledge

A very worrisome finding in the current study was the respondents’ low knowledge of heart attack or stroke warning symptoms. Two-fifths (40.7%) of the study participants were not aware of any heart attack symptoms, and only 8.5% could identify all symptoms. Chest pain was the most common known symptom (50.4%), which is close to that found in Beijing [23], but higher than that in Pakistan [12] and Nepal [22]. In contrast, a higher knowledge was reported by previous studies from North Ireland, Canada, Iran and Jordan, where chest pain was known by over 75% of respondents [14, 15, 17, 20]. Shortness of breath was recognized by 48.0% of the study population, which is consistent to that reported in North Ireland, Canada and Jordan [14, 15, 20], but higher than that in Pakistan and Nepal [12, 22]. Iranians showed a higher knowledge than Kuwaitis regarding shortness of breath [17]. ‘Pain in arms or shoulder’ was identified by 41.2% of respondents, which concurs with that reported in Canada and Jordan [15, 20], but higher than that reported by studies from North Ireland and Nepal [14, 22]. ‘Feeling weak, light-headed, or faint’ was recognized by 25.4% of participants, which is close to that reported in North Ireland and Nepal [14, 22], but higher than that in Canada [15]. Failure to know heart attack symptoms may increase the delay in seeking early medical care that could lead to a worse therapeutic outcomes. These findings underscore the urgent need to educate the Kuwaiti population to recognize the multiple symptoms of heart attack, since better knowledge would lead to earlier presentation to medical care, which might result in improvements in patients outcomes [10, 12].

The study participants’ knowledge of stroke symptoms was low, about half of respondents (47.8%) were not aware of any stroke symptoms, and only 10.4% could identify all symptoms. The commonest stroke symptoms recognized by respondents were ‘confusion or trouble speaking or understanding others’ (36.4%), followed by ‘numbness or weakness of the face, arm, or leg’ (34.7%), and ‘dizziness, trouble walking, or loss of balance or coordination’ (32.2%). These figures were higher compared to that reported in Australia, Canada and the Gulf countries [11, 15, 19], but lower than that in the USA [24]. The present findings emphasize the need for effective stroke education efforts in Kuwait. As was the case with heart attack symptoms, better knowledge would lead to early recognition of stroke symptoms, proper emergency action and reduced mortality [11, 15].

Respondents’ knowledge regarding the CVD risk factors was better than that for the warning symptoms of heart attack and stroke. The median (IQR) score for knowledge about the nine CVD risk factors was 7.0 (4.0) [moderate knowledge], nearly half of respondents (48.2%) identified eight or nine risk factors. It is possible that this better awareness about CVD risk factors is related to their significant representation in mass media campaigns as opposed to warning symptoms of heart attack and stroke. The commonest risk factors identified by over four-fifths of the study participants were smoking, obesity, unhealthy diet and physical inactivity. Respondents higher knowledge about these four risk factors may be related to their intensive representation in mass media campaigns as opposed to hypercholesterolaemia, hypertension, diabetes, stress, and family history of CVD, which were identified less frequently by the study population.

Smoking was identified as the most common risk factor in this study, which is consistent to that reported in Pakistan [16], but higher than that stated in previous studies from North Ireland, Canada, Iran, the Gulf countries, Jordan, South Africa, and Nepal [14, 15, 17, 19–22]. The current results indicate a better knowledge about unhealthy diet as a risk factor when compared to previous reports from Pakistan, North Ireland, Jordan, and South Africa [12, 14, 20, 21]. Nevertheless, the study participants show lower knowledge related to physical inactivity and obesity as risk factors when compared with the figures in Pakistan, North Ireland, Iran, Jordan, and Nepal [12, 14, 16, 17, 20, 22]. Stress as a risk factor was identified by 62% of respondents, which is close to that reported in Iran, Jordan and South Africa [17, 20, 21], but higher than that in Pakistan, North Ireland, Canada, and Nepal [12, 14, 15, 22]. Positive family history of CVD is more commonly recognized by the study participants in comparison with previous studies from Pakistan, North Ireland, Canada, Jordan, and South Africa [12, 14, 15, 20, 21]. The current results demonstrate that Kuwaitis have a better knowledge regarding hypercholesterolemia, hypertension, and diabetes as risk factors when compared to previous studies from Pakistan, North Ireland, Canada, Iran, the Gulf area, Jordan, South Africa and Nepal [12, 14, 15, 17, 19–22].

These present findings highlight the need for further improvement of the public knowledge regarding CVD risk factors, with more emphasis on diabetes, hypertension, hypercholesterolaemia and stress, which are highly prevalent among the Kuwaiti population. Hypertension, hypercholesterolaemia, and diabetes are established risk factors, and their adequate control is known to reduce the incidence of heart attack and stroke. The health message should promote positive health behaviours, and explain to the public that knowledge and action regarding reduction of risk factors are linked to reduced morbidity and mortality. Health care professionals must also ensure that they impart education to their patients. In Kuwait, CHD is the major cause of morbidity and mortality so CVD risk factors must be well known by the general population to avoid high death rate due to CVD [3, 4].

Although about half of the study participants have high knowledge of CVD risk factors, approximately one-fifth of respondents never had their blood pressure checked, about one-quarter had never undergone assessment of their blood cholesterol or glucose levels, and almost one-quarter of the hypertensive patients and over half of those with dyslipidaemia reported not to take medications. Furthermore, nearly one-fifth are smokers, about two-thirds are either overweight or obese; almost three-quarters reported to exercise 0–2 times/week or do not eat healthy food regularly; and about one-third are very stressful or stressful. This may be attributed to that respondents did not consider themselves at risk and did not want to change their lifestyle and to take preventive measures. This may be explained by the Health belief model [8]. The probability of recognizing oneself at higher risk increases when the existence of a risk factor is known. Nevertheless, individuals often underestimate their CVD risk [28]. These findings underscore the need for urgent emphasis on behaviour change interventions to promote positive health behaviours among the public in Kuwait.

In the present study, the median (IQR) overall CVD knowledge score was 10 (8) out of a possible 25, suggesting an overwhelming lack of knowledge among the public in Kuwait. The current findings are consistent with previous studies conducted in several countries [12, 14–16, 20, 22]. This may signify that the lack of awareness about CVD among the general public is a worldwide problem, and highlights the necessity for the design of more targeted educational programs and models to increase the public’s awareness regarding CVD [12, 14, 18, 20]. Although knowledge of diseases alone is inadequate for better healthcare outcomes, it is a recognized vital pre-requisite to change the individuals’ health attitudes, behaviours and lifestyle practices [8, 9], as most decisions regarding modifications of an individuals’ behaviours rely, in part, on their perceptions and knowledge that certain behaviours are associated with increased disease risk [29].

### Factors independently associated with CVD knowledge

In the current survey, gender, age, level of education, eating healthy diet, and family history of CVD were found to be significantly associated with CVD knowledge (p <0.05). Knowledge about CVD was significantly higher among females compared to males, which is consistent with the study conducted by Attachi et al. [25]. Nevertheless, this is in contrast with the findings of previous studies, which reported non-significant association between gender and CVD knowledge [12, 14, 20, 22, 23]. The higher awareness among females may be related to their less working hours compared to males, which provides them with more time to utilize the mass media such as radio, television and newspapers, and become more knowledgeable about CVD [25]. The current study showed that participants aged 50–59 years were more knowledgeable about CVD compared to other age groups. The significant association between age and CVD knowledge was reported by previous studies from Pakistan, China and Iran [12, 23, 25]. This is in contrast to other studies, which reported no significant difference in CVD knowledge among different age groups [14, 16, 20, 22]. The higher knowledge among the middle-aged Kuwaitis may indicate that that they have more opportunities and/or intention to access information about CVD. It was reported in the literature that cardiovascular health knowledge levels rise linearly until middle age, when the levels starts to plateau [30]. The low levels of knowledge among the younger age groups may indicate that schools in Kuwait do not have efficient and proper educational programs regarding CVD. As the years go by, they are likely to build up knowledge from the mass media.

Similar to previous surveys from Jordan, North Ireland and Pakistan, the present study revealed that CVD knowledge was significantly related to level of education, eating healthy diet, and family history of CVD [12, 14, 16, 20]. Respondents who attained high education had better knowledge than those attained low-intermediate education. This may be due to their more capability to comprehend health messages, which are delivered through the mass media. This finding highlights the need for targeting individuals with low-intermediate education with educational campaigns tailored to their level of understanding. The study participants who reported eating healthy diet everyday had better CVD knowledge. This is consistent with evidence from the literature that individuals who pay more attention to their diet had better CHD knowledge [14, 20]. Respondents who reported having a positive family history of CVD achieved higher CVD knowledge in comparison to those who did not. This supports the previous findings that individuals with a positive family history of CVD were more engaged in healthy behaviours and had better CHD knowledge [12, 14, 20, 31]. The current results revealed that the respondents’ monthly income, smoking status, weight and frequency of exercise were not significantly associated with CVD knowledge. This is in contrast to previous studies that reported low knowledge among those with low-income, smokers, high BMI, and those undertaking irregular exercise [14, 20, 31]. Furthermore, the current findings did not find a higher knowledge of CVD in respondents who have CHD or diabetes, hypertension and dyslipidemia (i.e., established CVD risk factors), indicating low cardiovascular health literacy even among those affected. This indicates that health care providers may not be targeting these individuals effectively about CVD, and highlights the need for more focus on these subjects who are at highest risk of stroke and/or heart attack.

### Role of pharmacist in CVD prevention and management

The present study revealed that most of respondents only identified the role that community pharmacists had to play is to help patients manage their medications, with a minimal role in providing advice on healthy lifestyle, or the measurement of blood pressure, blood cholesterol and glucose levels. These findings are similar to those reported by previous studies from North Ireland and Jordan [14, 20]. This can be due to the fact that the majority of community pharmacists in Kuwait are involved in counselling patients on health behaviours related to use of prescribed medications, but are less involved in counselling on personal health behaviours [32]. The low recognition of the pharmacist’s role to provide health promotion and education is unfortunate, since community pharmacists are the most accessible health care providers to the public, and in position to provide early detection of chronic diseases and to identify unhealthy lifestyles. Hence, they can assist patients to reduce CVD risk factors by education and counselling when appropriate, e.g., diet and weight management, regular exercise and smoking cessation [33]. Evidence is available in the literature for the roles of pharmacists in the prevention and management of CVD in primary care. These roles included provision of educational materials, screening and monitoring of blood pressure, blood glucose and blood lipids, interventions in areas such as smoking cessation, lifestyle modification, medicines management and medicines adherence [34, 35].

The high willingness shown by the respondents to use CVD disease prevention and management services if become available in the community pharmacy would certainly be an advantage to the provision of cardiovascular health promotion activities in pharmacies of Kuwait. Community pharmacies need to develop effective and accessible services, and to be promoted to the public. A joint sustained collaboration between the Ministry of Health, the Pharmaceutical and Medical Associations and Kuwait University is essential to design and implement effective professional service training programs towards sharpening pharmacists’ knowledge and practical clinical skills to provide consistent and evidence based cardiovascular health promotion services. The strong cooperation between the professional associations, faculty of pharmacy, continuing education centers, and practicing pharmacists contributed effectively in the development of community pharmacy services [36]. Pharmacists who effectively perform cardiovascular health promotion activities in Kuwait should be identified so that they may act as a role model for others.

The present findings allow for important comparative work with existing and future investigations in middle-eastern countries, and worldwide. Given that CVDs are the most common cause of death in Kuwait, the study results will help policy makers, health services to understand the current public knowledge, and to be able to design more wide-spread and effective educational interventions. These interventions should (i) focus primarily on the recognition of heart attack and stroke warning symptoms, which is an important prerequisite for prompt and effective care; (ii) provide further improvement of the population’s knowledge about CVD risk factors, particularly diabetes, hypertension, hypercholesterolaemia and stress; and (iii) be sensitive to the attitudes, perceptions and abilities of targeted individuals. These can be provided to the public via all media means and workshops. Primary care physicians should also impart education to their patients, as patients usually rely on physicians for information. Given proper education and training, community pharmacists can be important front-line contributors to promote cardiovascular health. Furthermore, a collaborative multi-disciplinary team approach, including primary care physicians, nurses and community pharmacists should be established to improve the CVD knowledge gap among the public in Kuwait.

### Strengths and limitations

The strength of this study is that we used appropriate sampling method and sample size to generate a representative data about the study population. Further strength is the high response rate. The results can, therefore, be generalized at the study population level in Kuwait. We acknowledge that this type of study, using a self-administered questionnaire, has its limitations. It depends very much upon information given by respondents and open to recall bias or error. The extent of truthful answers or verifying respondents’ claims is not possible in this type of study, which were taken at face value. A further limitation of the study is the cross-sectional nature of the data that represented one point in time and, therefore, do not reflect any changes in respondents’ Knowledge about CVDs.

## Conclusions

This is the first known study to demonstrate the baseline levels of knowledge about CVD types, warning symptoms of heart attack or stroke, and CVD risk factors among the general population in Kuwait. The study participants showed deficiencies in CVD knowledge, which could turn into insufficient preventative behaviours and suboptimal patient outcomes. The study findings underscore the urgent need to establish more wide-spread and effective educational interventions, which should be sensitive to the perceptions, attitudes, and abilities of targeted individuals.

## Electronic supplementary material

Additional file 1: **Questionnaire to determine the knowledge of cardiovascular disease and its risk factors among the public in Kuwait.** (DOCX 32 KB)

## Abbreviations

CVDs: Cardiovascular diseases
CVD: Cardiovascular disease
CHD: Coronary heart disease
CI: Confidence interval
GCC: Gulf Cooperation Council
IQR: Interquartile range.
