# Supplementary material for: Public knowledge of cardiovascular disease and its risk factors in Kuwait: a cross-sectional survey
Source: BMC Public Health. 2014 Nov 4;14:1131. doi: 10.1186/1471-2458-14-1131 (PMC4237772; doi:10.1186/1471-2458-14-1131)
Supplement: Supplementary file 1 — Additional file 1: Questionnaire to determine the knowledge of cardiovascular disease and its risk factors among the public in Kuwait. (DOCX 32 KB) [file 12889_2014_7237_MOESM1_ESM.docx]

**Questionnaire to determine the knowledge of cardiovascular disease and its risk factors among the public in Kuwait**

**A. Demographic and other Characteristics**

**PLEASE FILL IN OR TICK (√) THE APPROPRIATE ANSWER**

1. **Age (in years**):…………………………….
2. **Gender:** Male Female
3. **Marital status:** Single Married Divorced Widowed
4. **Educational level:** Less than high school High school Diploma University

Postgraduate studies

1. **Employment:** Unemployed Retired Housewife Student Professional

Self-employed Clerical

1. **Residence:** Capital Hawalli Farwanhiya Ahmadi Jahra Mubarak Al-Kabeer
2. **Monthly income:**  Less than 500 KD 500-1000 KD Greater than 1000 KD
3. **Personal health:** Excellent Very good Good Fair Poor
4. **Height (meters)** :………………………………..
5. **Weight (Kg)**:……………………………………….
6. **How would you describe your weight?** Underweight Normal Overweight Obese
7. **Are you a smoker?** Yes (currently smoker) No (never smoked)

 previously smoked (Ex-smoker)

***If you answered “Yes” or “NO” to Q12 skip to Q14***

1. **If you are a past smoker, when did you stop smoking?**

Less than 6 months ago in the last 6-12 months more than 12 months ago

1. **In a typical week how many days do you do at least 30 minutes of exercise? (such as walking, running, cycling, jogging)** 0-2 times 3-5 times 5 times or more
2. **How often do you eat healthy food? (Plenty of fruits and vegetables, foods low in saturated fat, cholesterol, salt and high in fiber)** Everyday Not everyday
3. **How do you describe your lifestyle?**

Very stressful Stressful Relatively Stressful Free from stress

1. **Have any of your immediate family members been diagnosed with a cardiovascular disease?**

**(Mother, father, sister, brother, own child)** Yes No

**B. Information about Your Medical Status**

**PLEASE FILL IN OR TICK (√) THE APPROPRIATE ANSWER**

**18. Do you suffer from any of the following chronic diseases?***

|  |  | Yes | No |
| --- | --- | --- | --- |
|  | Hypertension |  |  |
|  | Diabetes |  |  |
|  | High blood cholesterol level |  |  |
|  | Coronary heart disease |  |  |

***If you answered “YES” for any of the diseases in Q18, please answer Q19, but if you answered “NO” for all the diseases in Q18 skip to Q20***

**19. If yes for any of the diseases in Q18, do you take any medications for the disease(s)?**

|  |  | Yes | No |
| --- | --- | --- | --- |
|  | Hypertension |  |  |
|  | Diabetes |  |  |
|  | High blood cholesterol level |  |  |
|  | Coronary heart disease |  |  |

**20. Information about the recent measures of your blood pressure, blood cholesterol and blood glucose.**

|  |  | **Normal** | **High** | **I do not know** |
| --- | --- | --- | --- | --- |
|  | Your recent blood pressure |  |  |  |
|  | Your recent cholesterol level |  |  |  |
|  | Your recent fasting blood glucose level |  |  |  |

**21. When was the last time you checked your blood pressure, blood cholesterol, blood glucose and weight?**

|  |  | **Never being checked before** | **Unsure/**  **I do not know** | **Checked within the last 1-3 months** | **Checked within the last 4-6 months** | **Checked within the last 7-12 months** | **Checked more than**  **1 year** |
| --- | --- | --- | --- | --- | --- | --- | --- |
|  | Blood pressure |  |  |  |  |  |  |
|  | Blood cholesterol |  |  |  |  |  |  |
|  | Blood glucose |  |  |  |  |  |  |
|  | Body weight |  |  |  |  |  |  |

**C. Knowledge about Cardiovascular Diseases**

**Please tick (√) the box that most appropriately reflects your opinion**

**22. Which of the following do you think are types of cardiovascular diseases?**

|  |  | **Yes** | **No** | **I do not Know** |
| --- | --- | --- | --- | --- |
|  | Coronary heart disease |  |  |  |
|  | Cerebrovascular disease |  |  |  |
|  | Peripheral arterial disease |  |  |  |
|  | Rheumatic heart disease |  |  |  |
|  | Congenital heart disease |  |  |  |
|  | Deep vein thrombosis and pulmonary embolism |  |  |  |

**23. Which of the following do you think are symptoms of a heart attack?**

|  |  | **Yes** | **No** | **I do not Know** |
| --- | --- | --- | --- | --- |
|  | Pain or discomfort in the jaw, neck, or back |  |  |  |
|  | Feeling weak, light-headed, or faint |  |  |  |
|  | Chest pain or discomfort |  |  |  |
|  | Pain or discomfort in arms or shoulder |  |  |  |
|  | Difficulty in breathing or shortness of breath |  |  |  |

**24. Which of the following do you think are symptoms of a stroke?**

|  |  | **Yes** | **No** | **I do not Know** |
| --- | --- | --- | --- | --- |
|  | Sudden numbness or weakness of the face, arm, or leg |  |  |  |
|  | Sudden confusion or trouble speaking or understanding others |  |  |  |
|  | Sudden trouble seeing in one or both eyes |  |  |  |
|  | Sudden dizziness, trouble walking, or loss of balance or coordination |  |  |  |
|  | Severe headache with no known cause |  |  |  |

**25. Which of the following do you think can put someone at high risk of developing cardiovascular disease?**

|  |  | **Yes** | **No** | **I do not Know** |
| --- | --- | --- | --- | --- |
|  | Smoking |  |  |  |
|  | Unhealthy diet such as diets high in saturated fats, cholesterol and salt |  |  |  |
|  | Physical inactivity (lack of exercise) |  |  |  |
|  | Obesity |  |  |  |
|  | Stress |  |  |  |
|  | Positive family history of cardiovascular disease |  |  |  |
|  | High LDL Cholesterol levels |  |  |  |
|  | Hypertension |  |  |  |
|  | Diabetes |  |  |  |

**D. Possible roles of health care professionals other than medical doctors in prevention and management of cardiovascular disease**

**Please tick (√) the box that most appropriately reflects your opinion**

**26. In addition to the medical doctors’ role in helping people to reduce their risk of cardiovascular disease, would you consider visiting the nurse or pharmacist instead of your medical doctor for any of the services listed below?**

|  |  | **Pharmacist** | **Nurse** | **Unsure** |
| --- | --- | --- | --- | --- |
|  | Offer advice on healthy diet |  |  |  |
|  | Offer advice on exercise |  |  |  |
|  | Offer advice on smoking cessation |  |  |  |
|  | Measure blood pressure |  |  |  |
|  | Measure blood glucose |  |  |  |
|  | Measure blood cholesterol |  |  |  |
|  | Helping patients managing their prescribed medicine |  |  |  |

**27. If the following services are offered in the community pharmacy, do you intend to use them?**

|  |  | **Yes** | **No** | **Unsure** |
| --- | --- | --- | --- | --- |
|  | Blood pressure measurement |  |  |  |
|  | Blood glucose measurement |  |  |  |
|  | Blood cholesterol level measurement |  |  |  |
|  | Body weight measurement |  |  |  |
|  | Advice on health diet |  |  |  |
|  | Advice on exercise |  |  |  |
|  | Advice on smoking cessation |  |  |  |
